# Transcriptomic and Proteomic Characterizations of the Molecular Response to Blue Light and Salicylic Acid in *Haematococcus pluvialis*

**DOI:** 10.3390/md20010001

**Published:** 2021-12-21

**Authors:** Xiaodong Wang, Chunxiao Meng, Hao Zhang, Wei Xing, Kai Cao, Bingkui Zhu, Chengsong Zhang, Fengjie Sun, Zhengquan Gao

**Affiliations:** 1 School of Life Sciences and Medicine, Shandong University of Technology, Zibo 255049, China; 15110702040@stumail.sdut.edu.cn (X.W.); mengchunxiao@sdut.edu.cn (C.M.); 20410010842@stumail.sdut.edu.cn (W.X.); 21410010879@stumail.sdut.edu.cn (K.C.); 21410010901@stumail.sdut.edu.cn (B.Z.); 21410010898@stumail.sdut.edu.cn (C.Z.); 2 School of Pharmacy, Binzhou Medical University, Yantai 264003, China; hao073711@bzmc.edu.cn; 3 School of Science and Technology, Georgia Gwinnett College, 1000 University Center Lane, Lawrenceville, GA 30043, USA

**Keywords:** transcriptomics, proteomics, blue light, astaxanthin, fatty acid, heme, reactive oxygen species, cell wall, salicylic acid

## Abstract

*Haematococcus pluvialis* accumulates a large amount of astaxanthin under various stresses, e.g., blue light and salicylic acid (SA). However, the metabolic response of *H. pluvialis* to blue light and SA is still unclear. We investigate the effects of blue light and SA on the metabolic response in *H. pluvialis* using both transcriptomic and proteomic sequencing analyses. The largest numbers of differentially expressed proteins (DEPs; 324) and differentially expressed genes (DEGs; 13,555) were identified on day 2 and day 7 of the treatment with blue light irradiation (150 μmol photons m^−2^s^−1^), respectively. With the addition of SA (2.5 mg/L), a total of 63 DEPs and 11,638 DEGs were revealed on day 2 and day 7, respectively. We further analyzed the molecular response in five metabolic pathways related to astaxanthin synthesis, including the astaxanthin synthesis pathway, the fatty acid synthesis pathway, the heme synthesis pathway, the reactive oxygen species (ROS) clearance pathway, and the cell wall biosynthesis pathway. Results show that blue light causes a significant down-regulation of the expression of key genes involved in astaxanthin synthesis and significantly increases the expression of heme oxygenase, which shows decreased expression by the treatment with SA. Our study provides novel insights into the production of astaxanthin by *H. pluvialis* treated with blue light and SA.

## 1. Introduction

Astaxanthin (3,3′-dihydroxy-β-carotene-4,4′-dione) is a type of red-orange carotenoid with strong biological antioxidant capacity [1] and important applications in human health and in the nutraceutical, cosmetics, food, and feed industries [2]. During the process of astaxanthin synthesis, isopentenyl diphosphate (IPP) reacts with dimethylallyl diphosphate (DMAPP) to form geranyl pyrophosphate (GPP), which joins another molecule of IPP to form farnesyl pyrophosphate (FPP). FPP joins IPP to create geranylgeranyl pyrophosphate (GGPP). Two molecules of GGPP are connected to generate phytoene, which undergoes multiple dehydrogenation reactions to produce lycopene. Then, lycopene undergoes two reactions to produce β-carotene and ultimately to generate astaxanthin [3]. Compared with other species of microalgae, *Haematococcus pluvialis* has shown the highest accumulation of astaxanthin—up to 4% of dry cell weight (DCW) [4]. In the production of astaxanthin, *H. pluvialis* goes through two developmental stages, including the dividing stage (morphologically green, producing macrozooids or zoospores and microzooids) and the astaxanthin accumulating stage (morphologically red, producing palmella and hematocysts or aplanospores). In the astaxanthin accumulating stage, the green dividing cells of *H. pluvialis* are transformed into palmella, which further develop into red aplanospores, while cells of red aplanospores continue to produce astaxanthin [5]. As a type of secondary metabolite, astaxanthin is mainly accumulated once the cell divisions in the microalgae largely terminate [6]. When the cells of *H. pluvialis* stop dividing, both the chloroplast size and the chlorophyll content are reduced. Astaxanthin is generally deposited in cytoplasmic lipid droplets (CLD) of *H. pluvialis* [7]. The accumulation of astaxanthin could be caused by various types of environmental stresses, including high light intensities, low nitrogen, salt stress, and high temperature [8].

Due to its less heat dissipation with long life-expectancy and small size, the light-emitting diode (LED) has become an important factor in the microalgal industry [9]. Blue, green, and red lights are important for the growth and development of microalgae [10]. For example, blue light increases the cell size of algae, lipid accumulation, and growing period in *Chlamydomonas reinhardtii* [11,12], while green light of high intensities could significantly promote photosynthesis [13], and red light increases growth rates in *Dunaliella salina* and *Spirulina* [14]; meanwhile, a red LED at a relatively low intensity enhances cell growth in *H. pluvialis* [15]. Studies have shown that different light qualities increase the production of astaxanthin, biomass, and fatty acid content in *H. pluvialis* [16,17], while high-intensity blue light increases the accumulation of astaxanthin in *H. pluvialis* [18]. Carotenoids in microalgae primarily function as the accessory pigments in light-harvesting antenna by absorbing blue light that is inadequately absorbed by chlorophyll, thus improving light absorption. Furthermore, astaxanthin has been shown to help prevent damage to the photosynthesis system in algae [7].

Studies have shown that addition of chemical regulators enhances the yield of microalgal biomass and biological products [19]. As a type of important plant hormone and signal molecule, salicylic acid (SA) shows critical functions in plant growth, immunity, and development, which have been extensively investigated [20]. Specifically, SA is involved in the molecular regulation of metabolic response to both abiotic stresses, i.e., heat, cold, drought, heavy metal, salt stresses [21,22,23], and biotic stresses, e.g., a plant’s defense against pathogens [24]. Our previous studies on SA inductions at the transcriptional level identified a group of plant-specific transcription factor families (e.g., MYB, AP2/ERF, WRKY, and HSF) in *H. pluvialis* in response to the treatment with SA [25,26]. Furthermore, studies have shown that SA regulates the astaxanthin synthesis of *H. pluvialis* by scavenging free radicals [27]. Under stressed conditions, a large amount of triacylglycerol (TAG) is also accumulated in *H. pluvialis* [28]. Astaxanthin mostly exists in the form of esterification, while an astaxanthin monoester joins an astaxanthin diester to form liposomes in the cytoplasm [29]. Moreover, under stresses, most of the de novo fatty acids are assembled into TAGs, which are esterified into cytosolic liposomes, along with astaxanthin esters through an unknown mechanism [30]. Therefore, it is important and beneficial to further explore the synthesis of fatty acids in order to identify novel experimental strategies to improve the accumulation of astaxanthin in microalgae.

As a key biological technology, RNA-seq is an important tool which is applied to investigate differential gene expression in various model organisms. For example, RNA-seq technology provides de novo gene sequence and annotation analyses [31]. With the complete genome of *H. pluvialis* recently sequenced [32], the molecular mechanism regulating the accumulation of astaxanthin in *H. pluvialis* could be further elucidated based on RNA-seq analysis. Previous studies based on transcriptome sequencing have investigated the molecular metabolism of *H. pluvialis* in different environments, e.g., strong light [33], different light qualities [10], salt ions [3,34], CO_2_ [35], and trisodium citrate [36]. Furthermore, studies showed that the accumulation of astaxanthin in *H. pluvialis* was affected by pyruvate metabolism [37]. To date, transcriptome sequencing has become an essential method for studying the molecular metabolism of *H. pluvialis*. Furthermore, proteomic analysis has been commonly used to characterize the functions of various types of proteins in the development of plants and microalgae. Due to the large amount of protein sequence information available and the application of mass spectrometry in sensitive rapid protein identification, proteomic sequencing provides a novel approach for the analysis of complex functions in plants and microalgae.

In this study, we first investigated the response of *H. pluvialis*, also known as *H. lacustris* [38], to blue light, and further explored the synergistic effects of both blue light and SA stresses on the molecular response of *H. pluvialis*. We then identified and analyzed the differentially expressed genes (DEGs) of *H. pluvialis* under blue and white lights to explore the response and adaptation of *H. pluvialis* to the change of light wavelength. RNA-seq analyses were performed based on samples treated with white light (day 0) and blue light with or without the addition of SA (day 2 and day 7). Our results indicated that combined analysis of transcriptome and proteome sequencing was efficient in clarifying the metabolic mechanisms of astaxanthin production by *H. pluvialis*. Our study clearly demonstrated that the combined analysis of RNA-seq and proteomic sequencing comprehensively revealed the variations in the molecular mechanisms of *H. pluvialis* in response to different wavelengths of light and SA, further providing essential insights into the metabolic regulation involved in the synthesis of astaxanthin at molecular level. We noted that our study was unique, based on the following: firstly, we applied the combination of both the transcriptomic and proteomic analyses in our investigation of the molecular response of *H. pluvialis* to both blue light and SA. Furthermore, we applied a high-intensity blue light, whereas previous studies generally utilized either a low-intensity blue light or a white light of high intensity with SA to treat *H. pluvialis*, or different species, e.g., *Ulva prolifera* [23,39,40]. Moreover, we reported, for the first time, the differentially expressed genes (DEGs) and proteins (DEPs) involved in the heme pathway at the genomic level.

## 2. Results

### 2.1. Transcriptome Analysis

A total of 999,477,676 raw reads generated from RNA-seq were further processed with the adapters removed, yielding a total of 872,291,032 clean reads of 149.92 Gb of sequence data, including 24.02 Gb, 68.40 Gb, and 57.50 Gb derived from samples on day 0, day 2, and day 7, respectively (Table S1). Based on the de novo assembly, a total of 109,443 unigenes with the N50 value of 1193 bp were annotated based on the clean reads of the three groups of samples, ranging from 201 bp to 13,626 bp in size. The size distributions of these unigenes revealed that a total of 35,250 unigenes ranged between 200 and 300 bp, followed by a total of 16,338 unigenes ranging from 300 to 400 bp (Figure S2). In total, 21,504 (~19.65%) unigenes were annotated based on the SwissProt database, 27,965 (~25.55%) unigenes were identified in the non-redundant (nr) protein database, 26,248 (~23.98%) unigenes displayed significant similarities to known proteins in the Pfam database, and totals of 19,648 (~17.95), 24,798 (~22.66%), and 31,590 (~28.86%) unigenes were annotated in the Kyoto Encyclopedia of Genes and Genomes (KEGG), the Gene Ontology database (GO), and the EggNOG database, respectively. The top two species with the highest number of annotated unigenes were *Chlamydomonas eustigma* (4558 unigenes of ~16.3%) and *C. reinhardtii* (2782 unigenes of ~9.95%) (Figure S1). There were 24,798 unigenes annotated to 11,212 GO terms revealed in three categories of the GO database, i.e., molecular function, cellular component, and biological process. The top two GO terms in these three categories were the biological process and the oxidation–reduction process of the molecular function, cytoplasm, and nucleus of the cellular component category, and ATP binding and protein binding of the biological process category. A total of 19,648 unigenes were enriched in 6 and 19 KEGG metabolic pathways at level 1 and level 2, respectively. The results of enrichment analysis based on the KEGG database revealed that the top two categories of metabolic pathways with the largest numbers of unigenes annotated were the metabolism category (4440 unigenes) in the KEGG pathway at level 1 and the translation category (1717 unigenes) in the KEGG pathway at level 2 (Table S2).

### 2.2. Identification and Enrichment Analysis of DEGs and DEPs

**Figure 1:**
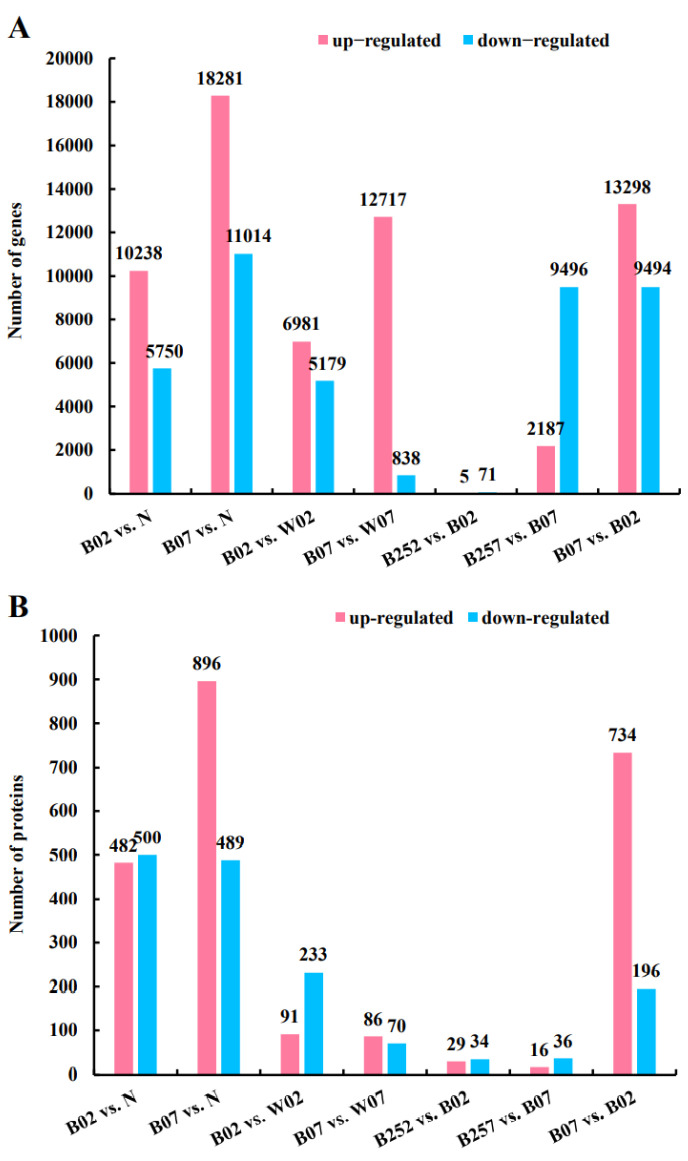
Differentially expressed genes (**A**) and proteins (**B**) identified among different pairwise comparisons of *Haematococcus pluvialis*.

As the treatment time increased from day 0 to day 7, the numbers of DEGs increased dramatically in the samples of *H. pluvialis*. The number of DEGs in the pairwise comparison of B257 vs. B07 was significantly higher than that of B252 vs. B02, suggesting that SA showed a weak effect on the microalgae in the early stage under the blue light culture and an enhanced effect in the late stage of the blue light treatment (Figure 1A). The most and the least numbers of DEGs of 29,295 and 76 were revealed in the pairwise comparisons of B07 vs. N and B252 vs. B02, respectively. As the treatment time of blue light irradiation increased, both the numbers of up-regulated and down-regulated genes increased significantly. In the groups treated with SA, there were only 76 DEGs, compared with the blue light group on day 2 of the treatment with blue light irradiation. However, on day 7 of the treatment with blue light irradiation, the numbers of up-regulated and down-regulated genes of the groups added with SA increased to 2187 and 9496, respectively (Figure 1A). The most and the least numbers of DEPs of 1385 and 52 were revealed in the pairwise comparisons of B07 vs. N and B257 vs. B07, respectively (Figure 1B).

To further investigate the effects of blue light and SA on *H. pluvialis*, we selected unigenes with significant changes in transcripts and proteins to perform the enrichment analyses based on both GO (Figure S3) and KEGG (Figure S4) databases (Table S3).

The results of the GO enrichment analysis showed that, on the second day of the treatment with blue light irradiation, most of the unigenes were annotated to carbon metabolism, e.g., glycolytic process, gluconeogenesis, and tricarboxylic acid cycle (Figure S3A). Notably, most genes, in response to cadmium ion, showed significant changes in their expressions. The results of the KEGG enrichment analysis showed that on the second day of the treatment with blue light irradiation, most of the unigenes were mainly enriched in metabolic pathways of energy metabolism, e.g., carbohydrate metabolism, metabolism of terpenoids and polyketides, and lipid metabolism (Figure S4A). On day 7 of the treatment with blue light irradiation, the unigenes showing significant changes in expression were mainly annotated to the GO terms related to proteins, e.g., translation, protein renaturation, and protein ubiquitin (Figure S3B), while the results of KEGG enrichment showed that the proportion of metabolic pathways of protein folding, sorting, and degradation was increased (Figure S4B). On the day 2 of the treatment with SA, no significantly expressed transcripts and proteins were observed. On day 7, with the addition of SA, most unigenes were annotated to GO terms of regulation of metabolic process, such as reactive oxygen species metabolic process, regulation of RNA metabolic process, and positive regulation by organization of apoptotic process (Figure S3C), while the results of KEGG enrichment analysis revealed that the significantly enriched metabolic pathways were related to infectious diseases (Figure S4C). These results were consistent with those reported previously, showing that SA, as one of the important signal molecules, played a vital role in disease resistance in plants [24].

### 2.3. DEGs and DEPs Involved in the Astaxanthin Metabolic Pathway

**Figure 2:**
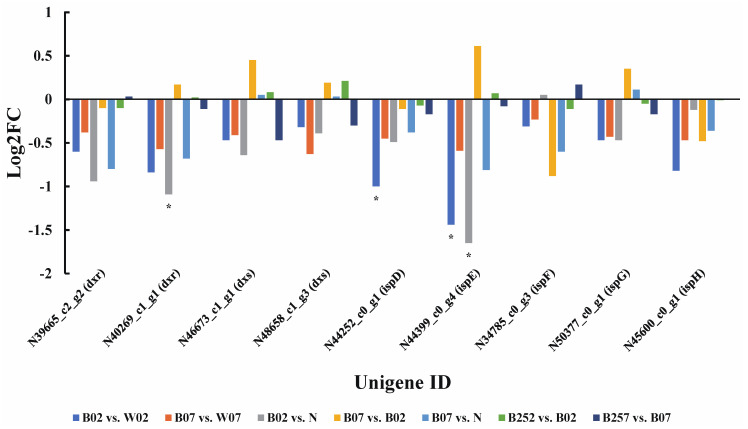
Expression profiles of the seven genes (i.e., *dxr*, *dxs*, *ispD*, *ispE*, *ispF*, *ispG*, and *ispH*) involved in MEP pathway based on transcriptomic analysis of pairwise comparisons in *Haematococcus pluvialis*. *—indicates significant down-regulation (*p* < 0.05 and Log2FC ≤ −1).

In *H. pluvialis*, isopentenyl diphosphate (IPP) is synthesized in the methyl-d-erythritol 4-phosphate (MEP) pathway [41] with a total of seven genes involved, including *dxr*, *dxs*, *ispD*, *ispE*, *ispF*, *ispG*, and *ispH* (Figure 2). In the early stage of exposure to blue light, the expression levels of *ispD* gene encoding 2-C-methyl-D-erythritol 4-phosphate cytidylyltransferase and *ispE* gene encoding 4-diphosphocytidyl-2-C-methyl-D-erythritol kinase were significantly lower than those treated with white light. Results showed that blue light showed little effect on the expression levels of *dxs* encoding 1-deoxy-D-xylulose-5-phosphate synthase, *dxr* encoding 1-deoxy-D-xylulose-5-phosphate reductoisomerase, *ispF* encoding 2-C-methyl-D-erythritol 2,4-cyclodiphosphate synthase, *ispG* encoding 1-hydroxy-2-methyl-2-(E)-butenyl 4-diphosphate synthase, and *ispH* encoding 4-hydroxy-3-methylbut-2-enyl diphosphate reductase (Figure 2).

**Figure 3:**
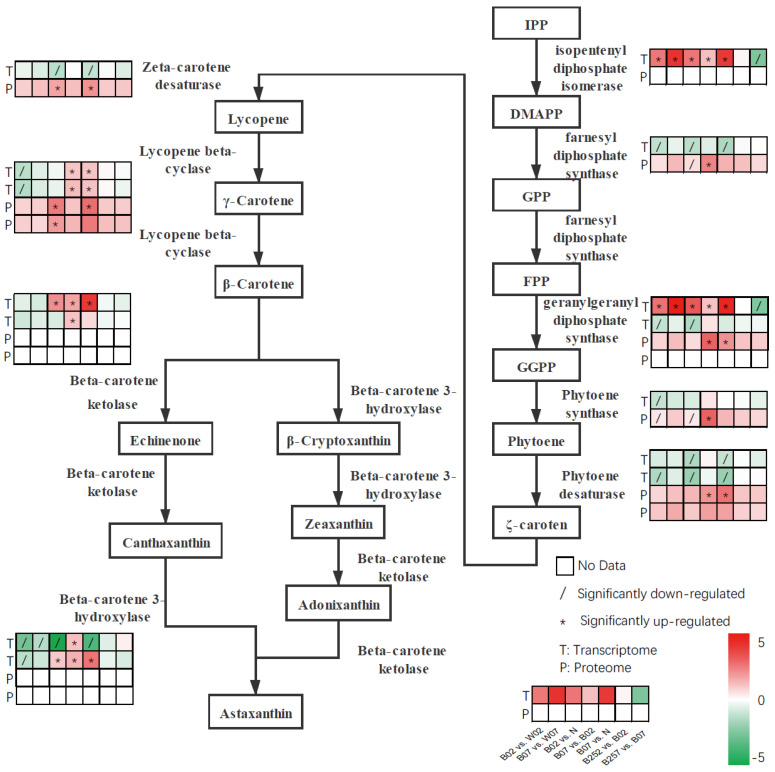
Expressions profiles of genes and proteins involved in astaxanthin synthesis pathway revealed in the pairwise comparisons in *Haematococcus pluvialis* based on transcriptomic and proteomic analyses.

The astaxanthin synthesis is initiated with the precursor of astaxanthin, i.e., β-carotene. The IPP produced in the MEP pathway is catalyzed by IPP isomerase (IPI) to generate DMAPP, which joins three IPP molecules to produce GGPP, catalyzed by farnesyl diphosphate synthase (FPPS) and GGPP synthase (GGPS). Subsequently, two GGPP molecules are catalyzed by phytoene synthase (PSY) to produce phytoene, which is used to generate ξ-carotene catalyzed by phytoene desaturase I (PDS). Then, ξ-carotene is used to generate lycopene, catalyzed by ξ-Carotene desaturase II (ZDS). Finally, lycopene cyclase (LCYB) catalyzes the transformation of lycopene into β-carotene, which is catalyzed by both beta-carotene hydroxylase (CRTR-B) and beta-carotene ketolase (BKT) to generate astaxanthin (Figure 3). Results of the transcriptome analysis showed that blue light exposure significantly up-regulated the expression of *ipi*, with its expression level escalated as the exposure time increased. However, on day 7 of the treatment with SA, the gene expression level of *ipi* was significantly decreased. On day 2 after exposure to blue light, compared with white light, the expression levels of *fpps*, *pds*, *psy*, and *crtR-B* (encoding β-carotene hydroxylase) were significantly decreased. On day 7 after the exposure to blue light, the expression level of *psy* was significantly increased, while the expression of *crtR-B* was significantly decreased. The addition of SA resulted in a significant decrease in the expression of *psy* on day 7 (Figure 3). On day 2 after the blue light exposure, the expression level of *crtR-B* was significantly down-regulated compared with that of *H. pluvialis*, treated with white light. However, the gene expression of *crtR-B* was increased as the exposure time increased. Blue light exposure showed no significant effect on the expression of gene *bkt* encoding β-carotene ketolase. However, with the increase in exposure time, the expression of *bkt* was significantly decreased.

### 2.4. DEGs and DEPs Associated with the Lipid Metabolic Pathway

When *H. pluvialis* is cultured under stressed environments, astaxanthin accumulation is usually accompanied by the synthesis of a large amount of fatty acids. TAGs are usually synthesized de novo from fatty acids and combined with astaxanthin to form liposomes [29]. As the precursor of fatty acid synthesis, NADPH is derived from the pentose phosphate pathway (PPP) with the involvements of both glucose-6-phosphate dehydrogenase (G6PD) and 6-phosphogluconate dehydrogenase (6PGD) [42].

**Figure 4:**
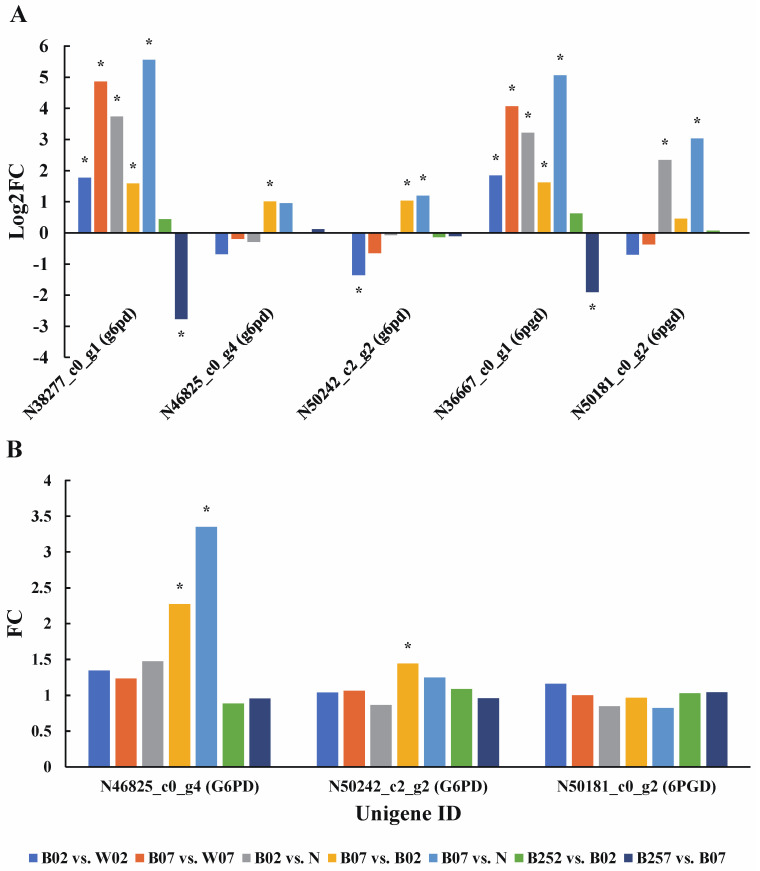
Expression profiles of genes *6pgd* and *g6pd* (**A**) and proteins 6PGD and G6PD (**B**) involved in the NADPH metabolism revealed in the pairwise comparisons of *Haematococcus pluvialis* based on transcriptomic and proteomic analyses, respectively. *—indicates significant up-regulation (*p* < 0.05 and Log2FC ≥ 1; *p* < 0.05 and FC ≥ 1.5) or significant down-regulation (*p* < 0.05 and Log2FC ≤ −1).

Our results showed that two and three unigenes were identified encoding 6PGD and G6PD, respectively (Figure 4A). Transcriptome analysis showed that on the second day of the treatment with blue light irradiation, the expression of *6pgd* was up-regulated, while the two transcripts of *g6pd* showed opposite expression patterns with one significantly up-regulated and the other significantly down-regulated. On day 7, the expressions of both 6PGD and G6PD were significantly up-regulated (Figure 4B). Compared with white light, no significant changes of both 6PGD and G6PD were observed under blue light irradiation on day 7. The addition of SA caused the down-regulation of the expression of both *6pgd* and *g6pd* on day 7. However, no significant changes caused by SA were observed at the protein level (Figure 4B).

**Figure 5:**
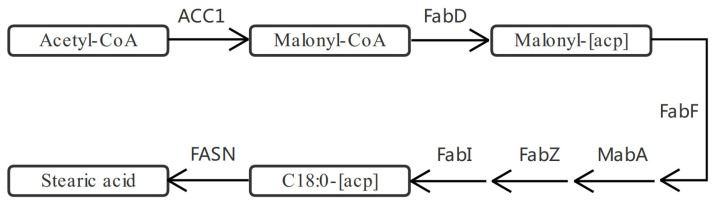
Fatty acid synthesis pathway in *Haematococcus pluvialis*.

In *H. pluvialis*, the fat acid synthesis is initiated with acetoyl-COA, which is catalyzed by acetyl-CoA carboxylase (ACC1) to synthesize malonyl-COA. The malonyl-COA reacts with acyl carrier protein (ACP) catalyzed by malonyl-CoA:ACP transacylase (FabD) to produce malonyl-[ACP] (Figure 5). After multiple reactions catalyzed by enzymes of beta-ketoacyl-[acyl-carrier-protein] synthase II (FabF), 3-oxoacyl-[acyl-carrier-protein] reductase (MabA), 3-hydroxyacyl-[acyl-carrier-protein] dehydratase (FabZ), and FabI, the C18:0-[ACP] is finally formed. Then, C18:0-[ACP] is catalyzed by fatty-acid synthase (FASN) to generate stearic acid [33].

**Figure 6:**
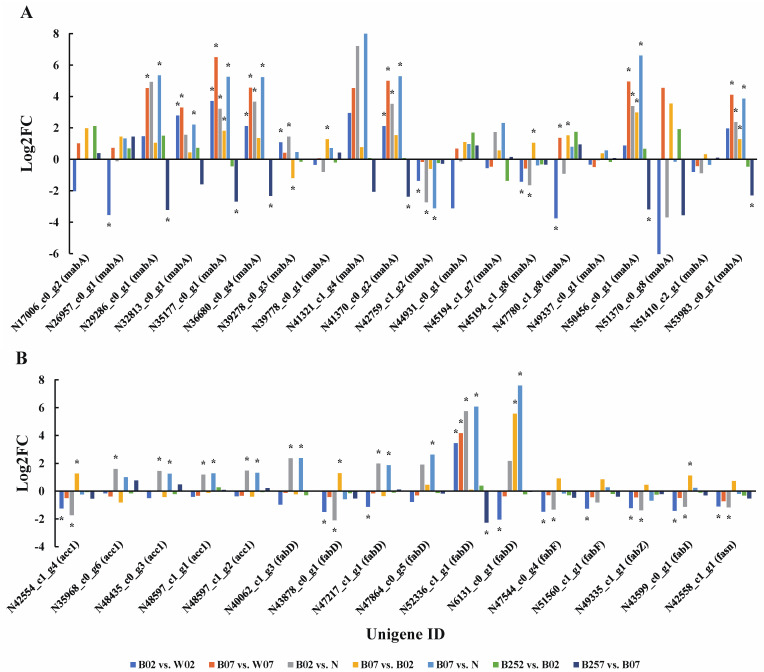
Expression profiles of genes *mabA* (**A**) and *acc1*, *fabD*, *fabF*, *fabI*, *fabZ*, and *fasn* (**B**) involved in the lipid metabolic pathway revealed in the pairwise comparisons of *Haematococcus pluvialis* based on transcriptomic analysis. *—indicates significant up-regulation (*p* < 0.05 and Log2FC ≥ 1) or significant down-regulation (*p* < 0.05 and Log2FC ≤ −1).

**Figure 7:**
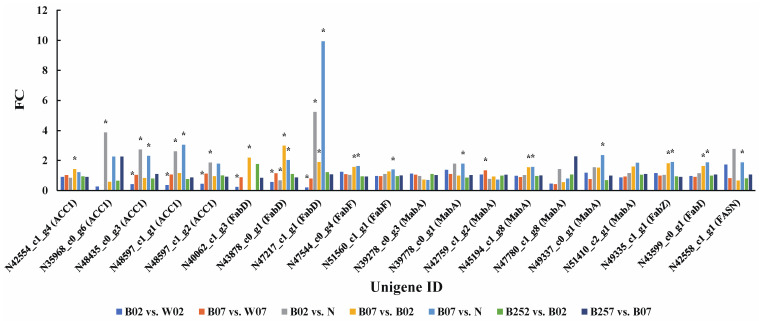
Expression profiles of proteins (i.e., ACC1, FabD, FabF, FabI, FabZ, FASN, and MabA) involved in the lipid metabolic pathway revealed in the pairwise comparisons of *Haematococcus pluvialis* based on proteomic analysis. *—indicates significant up-regulation (*p* < 0.05 and FC ≥ 1.5) or significant down-regulation (*p* < 0.05 and FC ≤ 0.67).

Compared with white light, on the second day of the treatment with blue light irradiation, five of the seven genes (i.e., *acc1*, *fabF*, *fabI*, *fabZ*, and *fasn*) related to fatty acid synthesis were significantly down-regulated, while the other two genes (i.e., *fabD* and *mabA*) showed transcripts either significantly up-regulated or down-regulated. On day 7 of the treatment with blue light irradiation, both *fabD* and *mabA* were significantly up-regulated. No significant changes were revealed in the early stage (day 2) of the treatment with SA, whereas both *fabD* and *mabA* were significantly down-regulated on day 7 (Figure 6). At the protein level, ACC1 and FabD were significantly down-regulated on the second day of the treatment with blue light irradiation, while only MabA was significantly up-regulated on day 7 (Figure 7). No significant changes were observed on these proteins with the addition of SA in *H. pluvialis*.

### 2.5. DEGs and DEPs Associated with the Heme Pathway

**Figure 8:**
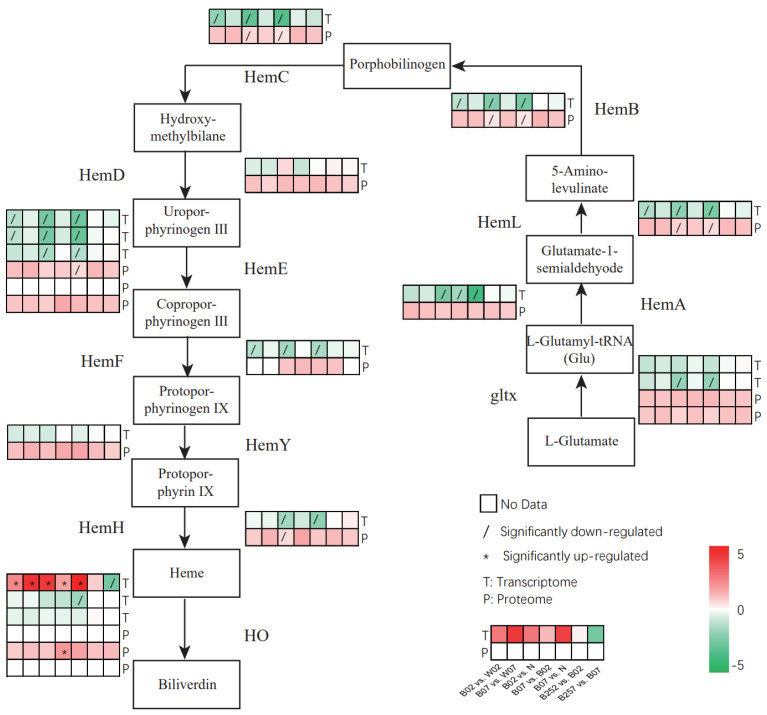
Expressions profiles of genes and proteins involved in the heme metabolic pathway revealed in the pairwise comparisons of *Haematococcus pluvialis* based on transcriptomic and proteomic analyses.

The heme metabolic pathway, with a total of 10 enzymes involved in *H. pluvialis*, was constructed based on the KEGG analysis (Figure 8). These enzymes included glutamyl tRNA synthetase encoded by *gltx*, glutamyl tRNA reductase encoded by *hemA*, glutamate-1-semialdehyde 2,1-aminomutase encoded by *hemL*, porphobilinogen synthese encoded by *hemB*, porphobilinogen deaminase encoded by *hemC*, uroporphyrin III synthese encoded by *hemD*, uroporphyrogen decarboxylase encoded by *hemE*, coprophyrinogen III oxide encoded by *hemF*, protoporphyrogen IX oxide encoded by *hemY*, and Ferrochealatase encoded by *hemH* (Figure 8). Heme is degraded by heme oxygenase encoded by *ho*. No significant changes were observed in the expressions of *hemD* and *hemY* in different treatment groups of *H. pluvialis*, while six genes (i.e., *hemL*, *hemB*, *hemC*, *hemF*, *hemE*, and *hemD*) showed the same expression pattern of significant down-regulation in three pairwise comparisons of B02 vs. W02, B02 vs. N, and B07 vs. N. The results of the KEGG analysis revealed two different transcripts of *gltx*, with one showing no significant change, while the other was significantly down-regulated in two pairwise comparisons of B02 vs. N and B07 vs. N. The proteomic results showed that the protein expression of HemL, HemB, and HemC were significantly down-regulated in two pairwise comparisons of B02 vs. N and B07 vs. N, while the addition of SA showed no significant effect on the expression of these genes and proteins. The expression of *ho* was significantly up-regulated by blue light, whereas the addition of SA resulted in the significant down-regulation of *ho* expression.

### 2.6. DEGs and DEPs Associated with the Generation of ROS

**Figure 9:**
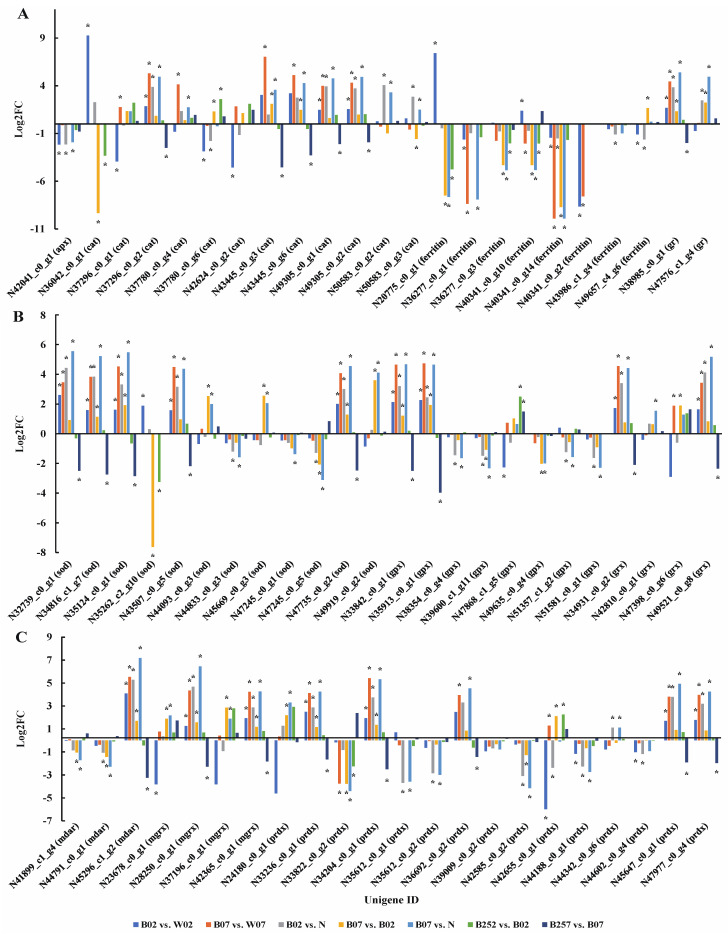
Expression profiles of genes—*apx*, *cat*, *ferritin*, and *gr* (**A**); *sod*, *gpx*, and *grx* (**B**); and *mdar*, *mgrx*, and *prdx* (**C**)—involved in the generation of ROS revealed in the pairwise comparisons of *Haematococcus pluvialis* based on transcriptomic analysis. *—indicates significant up-regulation (*p* < 0.05 and Log2FC ≥ 1) or significant down-regulation (*p* < 0.05 and Log2FC ≤ −1).

Studies have shown that *H. pluvialis* scavenged harmful ROS in cells by up-regulating genes involved in the removal of ROS [10]. Although the ROS is not beneficial for cell survival, it plays an important role in the production of astaxanthin [43]. Besides the generation of a large amount of astaxanthin, cells of *H. pluvialis* also prevent oxidative stress based on the involvements of a group of ROS-scavenging enzymes, including ascorbate peroxidase (APX), catalase (CAT), glutathione reductase (GR), peroxiredoxin (PRDX), monodehydroascorbate reductase (MDAR), ferritin, superoxide dismutase (SOD), glutathione peroxidase (GPX), glutaredoxin (GRX), and monothiol glutaredoxin (MGRX) [29,44]. Our results showed that on day 7, the expression levels of *sod*, *mdar*, *grx*, and *gr* in *H. pluvialis* treated with blue light were significantly higher than those treated with white light (i.e., pairwise comparisons of B02 vs. W02 and B07 vs. W07), the expression levels of *mgrx*, *cat*, and *gpx* under blue light were significantly higher than those in white light, whereas the expression of *apx* on day 2 after the exposure to blue light was significantly lower than that under white light (Figure 9). The expression level of gene encoding ferritin on day 7 of the treatment with blue light irradiation was significantly lower than that of white light. However, the expression patterns of *cat*, *ferritin*, *gpx*, *mgrx*, and *prdx* varied on day 2 after the treatment with blue light irradiation. On day 7 with SA added, the expression levels of *cat*, *gr*, *mdar*, *prdx*, and *sod* were significantly decreased. The expression levels of genes encoding ferritin and sod were decreased significantly on day 2 after SA was added. However, the expressions of related homologous genes were down-regulated. With the addition of SA, the expression levels of most ROS scavenging genes were decreased significantly and the down-regulated on day 7 after the treatment. Furthermore, the expression profiles of these genes were also changed with the treatment time of SA (Figure 9).

**Figure 10:**
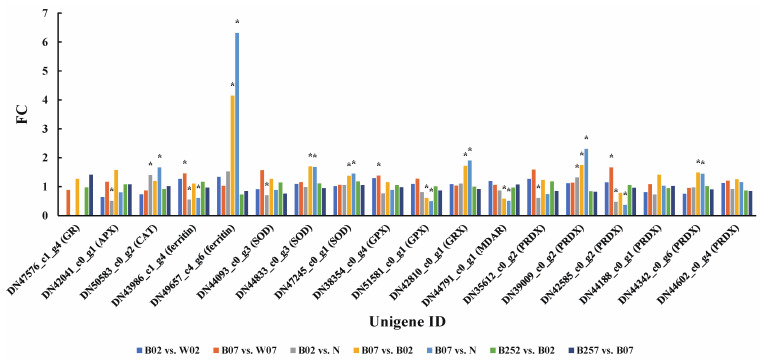
Expression profiles of proteins (i.e., APX, CAT, ferritin, GPX, GR, GRX, MDAR, PRDX, and SOD) involved in the generation of ROS revealed in the pairwise comparisons of *Haematococcus pluvialis* based on proteomic analysis. *—indicates significant up-regulation (*p* < 0.05 and FC ≥ 1.5) or significant down-regulation (*p* < 0.05 and FC ≤ 0.67).

A total of nine proteins related to ROS clearance pathway were detected based on the proteomic sequencing (Figure 10). On day 2 after the treatment with blue light irradiation, the expression levels of GPX, MDAR, and PRDX increased, whereas the expression levels of CAT, SOD, and PRDX decreased (Figure 10). On day 7 of blue light irradiation, the expression levels of ferritin and GPX were slightly increased, and the expression of PRDX was significantly increased. However, no significant effect of the addition of SA was observed on the expression of related proteins in the ROS clearance pathway. With the addition of SA, the genes related to ROS clearance pathway were significantly down-regulated at the transcriptional level, whereas no significant changes were observed at the proteomic level.

### 2.7. DEGs and DEPs Associated with the Cell Wall Biosynthesis

**Figure 11:**
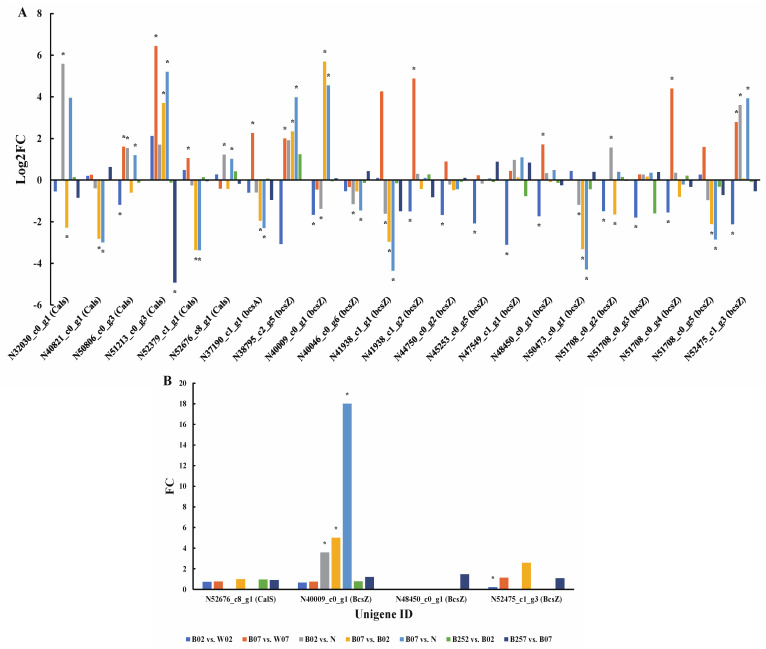
Expression profiles of genes *bcsA*, *bcsZ*, and *Cals* (**A**) and proteins BcsZ and CalS (**B**) involved in the cell wall biosynthesis revealed in the pairwise comparisons of *Haematococcus pluvialis* based on transcriptomic and proteomic analyses, respectively. *—indicates significant up-regulation (*p* < 0.05 and Log2FC ≥ 1; *p* < 0.05 and FC ≥ 1.5) or significant down-regulation (*p* < 0.05 and Log2FC ≤ −1; *p* < 0.05 and FC ≤ 0.67).

When *H. pluvialis* is cultured in stressed environments, the cell walls of the microalgae are thickened to enhance the capability of resisting adverse factors [45]. Callose, cellulose, and pectins are fundamental structural components of the cell wall and are synthesized with the involvement of many enzymes including callose synthase (CalS), endoglucanase (BcsZ), and cellulose synthase (BcsA) [44]. In our study, a total of 6 unigenes encoding CalS, 1 unigene encoding BcsA, and 15 unigenes encoding BcsZ were identified. The expression levels of genes encoding CalS and BcsZ were decreased significantly on day 2 of the treatment with blue light irradiation compared with white light (Figure 11A). On day 7 of blue light irradiation, the expression levels of *Cals*, *bcsZ*, and *bcsA* were significantly higher than those under white light. The addition of SA did not significantly affect the expression levels of genes encoding CalS and BcsZ but significantly down-regulated the expression of the genes encoding CalS on day 7 of the treatment. The proteomic data showed that the expression of BcsZ was decreased significantly on day 2 after the treatment with blue light irradiation compared with white light. These results were consistent with those derived from the transcriptomic data. Compared with day 0, the expression of BcsZ was significantly increased on both days 2 and 7 of the treatment with blue light irradiation, indicating that the expression of BcsZ was less affected by blue light than by white light (Figure 11A). These results were consistent with those derived from the proteomic data (Figure 11B).

### 2.8. DEGs Associated with the Blue Light Receptors

Plants utilize photoreceptors to regulate growth and development under light of different wavelengths. Both cryptochrome (CRY) and phototropin (PHOT) are blue light receptors, with conserved signal transduction in different plants. Members of eukaryotic CRYs include plant CRYs (pCRY), animal CRYs (aCRY), and *Drosophila*, *Arabidopsis*, *Synechocystis*, and Human (DASH) CRYs (dashCRY) [46,47]. Four CRYs (i.e., one pCRY, one aCRY, and two dashCRYs) were identified in *C. reinhardtii* [48] and seven CRYs (i.e., one pCRY, one aCRY, and five dashCRYs) were found in *Volvox carteri* [49]. The gene encoding pCRY contains multiple copies in higher plants but a single copy in eukaryotic green algae. To investigate the molecular mechanism regulating the astaxanthin accumulation in response to the blue LED, the CRYs were chosen for further transcriptomic analysis. In our study, seven CRYs were identified in *H. pluvialis* (Table 1). As the exposure time of blue light increased, the expression levels of genes encoding dashCRYs and CRYs were significantly decreased on day 2 and day 7 compared with those of day 0. Two genes encoding PHOT showed different expression patterns, with one showing no significant change and the other significantly down-regulated. The expression of one gene encoding dashCRY was significantly decreased from day 2 to day 7. On day 7 of the treatment with blue light exposure, the expression levels of genes encoding all blue light receptors were decreased compared with those of white light exposure. No significant changes in the expression of the genes encoding dashCRYs were observed with the addition of SA.

### 2.9. Identification of Transcription Factors

Transcription factors (TFs) are important for the growth, development, and reproduction of organisms [50]. Understanding the metabolic response of TFs to the treatments with blue light and SA helps further investigate the molecular mechanism regulating both the astaxanthin and lipid synthesis. Plants improve their tolerance to a variety of stresses with the involvement of TFs, which in turn are affected by a variety of molecular factors [51]. In our study, a total of 54 TF families were identified in *H. pluvialis* based on the transcripts assembled de novo using iTAK. The top 10 TF families included C2H2 with 94 unigenes, TRAF (90 unigenes), SNF2 (75 unigenes), C3H (67 unigenes), GNAT (59 unigenes), SET (54 unigenes), MYB (48 unigenes), zn-clus (46 unigenes), MYB-related (44 unigenes), and HMG (42 unigenes). Results of the comparative transcriptomic analysis of the control and the experimental groups of *H. pluvialis* treated with blue light irradiation revealed a total of 503 putative genes assigned in 47 families encoding TFs in response to blue light exposure (Table S4). Compared with white light, the expression levels of genes encoding two TF families (i.e., SNF2 and zn-clus) were significantly increased on day 2 of the treatment with blue light irradiation.

Varied compositions of the dominant TFs were also revealed under different treatments of *H. pluvialis* (Table 2). For example, there was a lack of GNAT and MYB families of TFs on day 2 and day 7 after the treatment with blue light, respectively. On day 7 with the addition of SA, the TFs of the jumonji family were not identified. Studies have shown that these TFs play important roles in various biological processes. For example, zinc finger proteins (e.g., C2H2) regulate various cellular processes [52], TRAF induces the activation of several kinase cascades—eventually leading to the activation of signal transduction pathways and regulation of various cellular processes [53], and SNF2 regulates the structure of chromatin and participates in biological processes, such as cell differentiation and immune response [54]. As the potential regulators in the astaxanthin synthesis of *H. pluvialis* under blue light, the functions of these families of TFs identified in our study warrant further investigations based on combined genetic, molecular, and biochemical approaches.

## 3. Discussion

### 3.1. Synthesis of Astaxanthin and Fatty Acids under Blue Light Irradiation

FPPS is a key enzyme in isoprenoid biosynthesis [55], providing the precursors (i.e., sesquiterpene) for carotenoids. Our results showed that the expression of *fpps* was significantly regulated on the second day of blue light irradiation. Then, the flow of precursors of astaxanthin synthesis was reduced. Both PSY and PDS are the rate-limiting enzymes in the carotenoid biosynthesis pathway of *H. pluvialis* [56,57]. Furthermore, both BKT and CRTR-B are involved in the synthesis of astaxanthin in *H. pluvialis* [58]. Therefore, these four enzymes are essential for the synthesis of astaxanthin in *H. pluvialis*. Previous studies showed that, compared with white light of 150 μmol photons m^−2^s^−1^, blue light of 70 μmol photons m^−2^s^−1^ significantly increased the expression of *psy*, *lcy*, *bkt*, and *crtR-B* in *H. pluvialis* [9]. Our results showed that blue light reduced the expression of genes involved in astaxanthin synthesis in early stage of blue light irradiation, probably due to the different light intensities, suggesting that high-intensity blue light was not conducive for astaxanthin synthesis. It was expected that the carotenogenesis was correlated with the patterns of gene and protein expressions revealed in the current study. Future complementary studies are necessary to investigate the contents of astaxanthin and other important metabolites produced in *H. pluvialis*, i.e., carotenoids, chlorophylls, amino acids (i.e., glutamate), and lipids. Moreover, both the qRT-PCR and genetic knockout experiments are required to verify the expression patterns and functions of genes and proteins involved in astaxanthin biosynthesis pathway with the goals to explore the molecular mechanisms underlying the production of astaxanthin in *H. pluvialis* treated with high-intensity blue light and SA.

Previous studies showed that high light irradiation enhanced the synthesis of fatty acids in *H. pluvialis*, and the gene expression was significantly up-regulated with the increase in treatment time of high light irradiation [3,29]. However, our results showed that on the second day of the treatment with blue light irradiation, both gene and protein expressions involved in the production of acetyl-CoA and NADPH were significantly down-regulated, and the genes and proteins directly involved in the fatty acid synthesis were also significantly down-regulated, ultimately leading to a decrease in fatty acid synthesis. However, no significant changes were observed in these genes on day 7 of the treatment with blue light irradiation. Compared with day 7, the shorter treatment time (day 2) of blue light irradiation showed a greater effect on the production of astaxanthin of *H. pluvialis*, specifically resulting in the significant down-regulation of genes involved in astaxanthin synthesis (Figure 3). These results were consistent with those of the changes in gene and protein expressions involved in the biosynthesis of fatty acids (Figure 10 and Figure 11). Because astaxanthin is esterified with fatty acids, the content of fatty acids is regulated with the change of astaxanthin content [59].

ROS burst in *H. pluvialis* was observed at the beginning of the treatment of light irradiation [44]. It is important for the cells of *H. pluvial is* to remove a large amount of ROS, which is also required in the production of astaxanthin. However, our results showed that many key genes involved in astaxanthin synthesis were significantly down-regulated on the second day of treatment with blue light irradiation, and genes in the fatty acid synthesis pathway were also significantly down-regulated, indicating that in the early stage of irradiation (day 0), the effect of blue light on astaxanthin production by *H. pluvialis* is less than that of white light. In the later stage of irradiation (day 7), blue light increased the astaxanthin production compared with white light. In the early stage of treatment (day 2), SA showed no significant effect on astaxanthin and fatty acid synthesis, while in the later stage (day 7), gene expression was significantly down regulated, probably because SA could regulate a variety of plant metabolic processes and the production of a variety of secondary metabolites to protect plants from abiotic stresses [22]. SA may also activate other defense pathways. For example, studies have shown that high light intensity and SA treatment for two days causes significant down-regulation of genes involved in astaxanthin and fatty acid synthesis [33], suggesting that *H. pluvialis* activates other metabolic processes to resist excessive ROS to adapt to the stressed environment. These results are consistent with our findings, showing that the gene expression was down-regulated on the 2nd day of blue light irradiation and on the 7th day of SA treatment.

### 3.2. Scavenging of Reactive Oxygen Species under Blue Light Irradiation

In general, organisms synthesize 5-ALA in one of the two ways, i.e., the C4 pathway and the C5 pathway. The C4 pathway mainly exists in animals and fungi, while the C5 pathway is utilized by plants, algae, and bacteria [60]. The 5-ALA is the precursor of chlorophyll, heme, and vitamin B12 biosynthesis pathways, with chlorophyll and heme synthesis pathways sharing several intermediate products. Our results showed that expression levels were decreased in genes (i.e., *hemL*, *hemB*, *hemC*, *hemD*, and *hemF*) involved in the shared portions between chlorophyll and heme synthesis pathways, whereas the expression of *ho* was significantly increased under blue light conditions (Figure 8). As the cofactor of catalase and many types of peroxidases, the increase in heme content removes excessive ROS produced in cells. Although the oxidation effect of the free heme damages cells under oxidative stress, cells develop a variety of protection mechanisms [61]. For example, studies have shown that heme oxygenase is involved in the antioxidant mechanism by catalyzing the degradation of heme to produce biliverdin, which shows antioxidant effect. The other two end products derived from biliverdin, i.e., CO and Fe/FTH, have also shown cytoprotective effect [62]. Compared with white light, the expression of heme oxygenase in blue light treatment group was significantly increased, leading to the increase in heme degradation products and the production of antioxidants. However, the expression of *ho* was decreased significantly on day 7 after the treatment with SA (Figure 8). As an important signal molecule, SA regulates a variety of oxidative stress responses to improve the efficiency of antioxidant system in plants. In *H. pluvialis*, a large amount of astaxanthin is generated to reduce the damage caused by the detrimental environment, complementing, or ultimately replacing, the antioxidant system of the microalgae. Therefore, *H. pluvialis* protects itself from the adverse effects of stress by accumulating more astaxanthin [29]. Our previous studies demonstrated that the addition of SA increased the astaxanthin content in *H. pluvialis* [4], suggesting that astaxanthin complements the antioxidant effect of heme degradation products and reduces the expression of *ho*. Similarly, in the blue light treatment groups, the expression of genes involved in astaxanthin synthesis (i.e., *psy*, *pds*, *lcyb*, and *crtr-b*) was also significantly down-regulated (Figure 6), whereas the expression of heme oxygenase was significantly up-regulated (Figure 8), suggesting that heme degradation products help maintain the intracellular homeostasis in *H. pluvialis*.

Previous studies showed that scavenging ROS was essential for the survival of *H. pluvialis* under high light intensity [63]. Our results showed that the expression levels of most genes related to ROS in *H. pluvialis* treated with blue light were significantly higher than those in white light, indicating that the damage of blue light to *H. pluvialis* was higher than that of white light (Figure 9). Previous studies showed that APX, CAT, GRX, GPX, and PRDX removed hydrogen peroxide [64], while SOD scavenged superoxide to form hydrogen peroxide [65]. Studies have shown that the antioxidant systems in different species of plants respond differently to the treatment with SA. For example, the activities of ascorbic APX and SOD were enhanced, and the activities of CAT were reduced in corn, whereas the activities of antioxidant enzymes CAT, peroxidase (POX), SOD, and glutathione reductase were decreased in rice [66]. Moreover, the level of H_2_O_2_ and the activities of antioxidant enzymes CAT, POX, and SOD were enhanced [66]. Our results showed that the treatment with SA led to the significant down-regulation of many genes involved in the ROS clearance pathway (Figure 9), suggesting the overall adjustment of resistance to stress in *H. pluvialis*. Furthermore, our previous studies showed that the addition of SA increased the content of astaxanthin in *H. pluvialis* [4]. These results suggested that SA reduced the expression of ROS scavenging genes, resulting in the production of a large amount of astaxanthin to maintain the homeostasis in cells of *H. pluvialis*.

### 3.3. Cell Wall Synthesis Inhibited in the Early Stage of the Treatment with Blue light Irradiation

Studies have shown that plants develop resistance to light stress of high intensity by regulating the composition of cell wall [67], while the light intensity and quality can affect the structure of cell wall [68]. For example, light regulates the sugar content of cell wall in peas [69]. Furthermore, studies have revealed that blue light showed a rapid inhibitory effect on the cell wall synthesis, while red light showed a long-term inhibitory effect in the pea seedlings [70]. Compared with white light, genes involved in cell wall synthesis were significantly down-regulated on day 2 but then significantly up-regulated on day 7 of the treatment with blue light irradiation, indicating that blue light showed less effect on *H. pluvialis* than that of white light in the early stage of blue light irradiation (day 2) but greater effect of long-term irradiation (day 7) on *H. pluvialis* than that of white light. These results were consistent with those derived from the proteomic data (Figure 11B). Furthermore, these results also suggested that the blue light inhibited the cell wall synthesis of *H. pluvialis* in the early stage of the treatment with blue light irradiation.

### 3.4. Signal Transduction of Blue Light Receptors

PHOT is activated by blue light to participate in physiological processes such as chloroplast movement and plant phototropism [71]. PHOT contains both the serine/threonine kinase binding domains at the C-terminal and two light, oxygen, and voltage (LOV) domains at the N-terminal [72]. Exposure of LOV to blue light causes cysteine residues in the protein to covalently bind to the isopyrazine ring to generate conduction signal [73]. In *Arabidopsis*, PHOT regulates the transcripts in response to high-intensity blue light, though PHOT is not largely involved in transcriptional regulation [74,75]. However, PHOT causes significant changes in expression of genes involved in chlorophyll and carotenoid biosynthesis in *C. reinhardtii*, whereas the low-intensity blue light leads to a significant increase in the expression of *pds* [71]. Furthermore, the expression of *pds* is inhibited as the expression of *phot* is inhibited [71]. In our study, the expression of *phot* was decreased, but not significantly, under blue light, compared with white light. In the chlorophyll synthesis pathway (Figure 8), most genes were significantly down-regulated on the second day but not on day 7 of the treatment with blue light irradiation. In the astaxanthin synthesis pathway (Figure 6), most genes were significantly down-regulated on day 2 of the treatment with blue light irradiation, while no significant changes were observed on day 7.

The C-terminal domain of CRY is responsible for providing the interaction sites for DNA or downstream signal proteins, while the function of the N-terminal domain is to combine the second chromophore as an optical collection antenna [76]. Based on knockdown mutants, studies have shown that CRY regulated the transcriptional levels of various genes under blue light, caused by its oxidized fad chromophore. Under the blue light irradiation, the C-terminal domain interacts directly with downstream signal proteins, e.g., ubiquitin ligase. The photolyase homologous region (PHR) of cryptochrome can form homodimers, which are also essential for signal transduction [77].

The cryptochrome (CPH1) protein of *C. reinhardtii* undergoes photoinduced degradation under white, red, and blue lights [48]. Similar results were also observed in our study, showing that the blue light irradiation of high intensity significantly reduced the expression of *cry* in *H. pluvialis*. Furthermore, CPH1 also functions as a transcription regulator to regulate gene expression and induce the expression of both chlorophylls a and b binding protein and *hemL* [48]. Our results showed that the expression of *hemL* was significantly down-regulated on the second day of the treatment with blue light irradiation, while no significant down-regulation was observed on day 7, and *cry* was not significantly down-regulated under high-intensity blue light.

Plants respond to light through photoreceptors. Our study showed that the expression of blue light photoreceptors (i.e., PHOT and CRY) were not significantly down-regulated under blue and white light irradiations of high intensity, suggesting that in *H. pluvialis*, the metabolic pathways are not affected by the content of photoreceptors but by the signal transduction of photoreceptors. For example, ROS at high concentrations causes damage in cells but functions as signal molecules at low concentrations. Activated CRY can produce trace ROS as signal molecules in response to light. Furthermore, the high content of CRY causes the increased production of ROS in response to light in *Arabidopsis* [78]. Our results showed that, compared with normal light intensity, high-intensity blue light significantly inhibited the expression of CRYs, while the expression of PHOTs was decreased on day 7 of the treatment of high-intensity irradiation. Studies have revealed significant up-regulation of CRY and PHOT in *Dunaliella salina* treated with blue light irradiation of 50 μmol photons m^−2^s^−1^, whereas no significant changes were observed with the treatment with blue light with an increased intensity of 150 μmol photons m^−2^s^−1^ [79]. This is probably because that low-intensity blue light was sufficient enough to activate the photoreceptors [80]. It is speculated that, after the light intensity increases to a certain level, the effect of light quality on the function of blue light receptors is reduced.

## 4. Materials and Methods

### 4.1. Algal Materials

*Haematococcus pluvialis* strain 712 (FACHB-712) obtained from the Freshwater Algae Culture Collection at the Institute of Hydrobiology (FACHB) was cultured in 5000 mL beakers, containing BBM medium without aeration, under a light intensity of 25 μmol photons m^−2^s^−1^ with a photoperiod of 12 h/12 h light/dark at 22 °C. When the cell growth reached the logarithmic phase (10^5^ cells mL^−1^), the culture was evenly divided into 9 aliquots each of 1000 mL. Samples were divided into 3 groups, including the control group grown under constant white light of 150 μmol photons m^−2^s^−1^ at 22 °C (W0 group), the group treated with constant blue light of 150 μmol photons m^−2^s^−1^ at 22 °C (B0 group), and the group pre-treated with SA (2.5 mg/L) under constant blue light of 150 μmol photons m^−2^s^−1^ at 22 °C (B25 group). The selection of the concentration of SA (2.5 mg/L) was based on the previous study [81] and our pre-experiments, which showed that the production of astaxanthin was increased with reduced concentration of SA. Each group contained three replicates.

### 4.2. Transcriptome Sequencing

The three groups of microalgal samples were collected on day 0 (under white light) and after treatment on both 48 h (day 2) and 168 h (day 7) of blue light and SA. The sample collected on day 0 was named N. Other samples were named by group names and treatment times (day). Specifically, B02 and B07 represented samples treated with blue light irradiation for 2 and 7 days without SA, respectively. W02 and W07 represented samples treated with white light irradiation for 2 and 7 days without SA, respectively. B252 and B257 represented samples treated with blue light irradiation for 2 and 7 days with the addition of SA (2.5 mg/mL), respectively. Total RNA was extracted from *H. pluvialis* (10 mL) using Trizol Reagent (Invitrogen, Carlsbad, CA, USA). The algal sample (30~50 mg) was first ground in liquid nitrogen (less than 100 mg), then 1000 μL Trizol Reagent was added, and grinding continued until homogenized. The homogenate was transferred to a small tube and set still for 10 min before being centrifuged for 5 min at 12,000 g and 4 °C. The supernatant was collected and set still for 5 min, then added with 200 μL of chloroform, shaken vigorously for 15 sec, and kept for 3 min at room temperature. Then, the sample was centrifuged for 15 min at 12,000 g and 4 °C. The water phase was transferred, twice the volume of isopropanol was added, it was vortexed briefly, and kept at −20 °C overnight. Then, the sample was centrifuged for 15 min at 12,000 g and 4 °C. The supernatant was removed, and the sample was added to 1 mL ethanol (70%) and centrifuged for 5 min at 7500 g and 4 °C. The supernatant was removed, and the ethanol was removed from the sample, which was set to dry for 7 min at room temperature. Then, 50 μL of water was added to the centrifuge tube to dissolve the sample, which was then vortexed to mix well. The RNA integrity was assessed using 1% formaldehyde denaturing agarose gel electrophoresis. Then, mRNA was purified from total RNA by deploy-t with magnetic beads. The RNA was processed by mRNASeq sample preparation kit (Illumina, San Diego, CA, USA) to establish the sequencing system. Transcriptome sequencing was performed using Illumina Hiseq 4000 (LC Sciences, Houston, TX, USA).

### 4.3. De Novo Assembly and Unigene Annotation

Cutadapt [82] and in-house Perl scripts at Hangzhou Lianchuan Biotechnology Co. Ltd. (LC Sciences, Houston, TX, USA), were used to remove adaptor sequences and low-quality bases. The sequence quality was evaluated by FastQC (https://www.bioinformatics.babraham.ac.uk/projects/fastqc/, accessed on 16 March 2020). The de novo assembly of transcriptome from *H. pluvialis* was performed using Trinity2.4.0 [83]. All assembled unigenes were aligned against the non-redundant (nr) protein (http://www.ncbi.nlm.nih.gov/, accessed on 16 March 2020), Gene Ontology (GO) (http://www.geneontology.org/, accessed on 16 March 2020), SwissProt (http://www.expasy.ch/sprot/, accessed on 16 March 2020), Kyoto Encyclopedia of Genes and Genomes (KEGG) (http://www.genome.jp/kegg/, accessed on 16 March 2020), and EggNOG (http://eggnogdb.embl.de/, accessed on 16 March 2020) databases using DIAMOND [84] with a threshold of E value < 0.00001. Transcription factors were identified using iTAK [85]. The selection of de novo assembly over genome-based analysis was based on two lines of evidence. First, the results of several biosynthetic pathways based on the genome-based analysis showed variations in the expression patterns of genes from those based on the de novo assembly. Therefore, we further investigated the cause of these inconsistent results by conducting the qRT-PCR analysis of genes involved in these biosynthetic pathways. Our results of qRT-PCR confirmed the expression patterns of genes based on the de novo assembly, whereas the results based on the genome-based assembly were largely not confirmed by qRT-PCR analysis. Second, our transcriptomic sequencing results based on the alignment program HISAT2 (https://daehwankimlab.github.io/hisat2/, accessed on 16 March 2020) revealed the overall alignment rate of only ~50% with the reference genome of *H. pluvialis* deposited at the NCBI database (GenBank accession number GCA_011766145.1 of BioProject PRJDB8952).

### 4.4. Differentially Expressed Unigenes Analysis

Salmon [86] was used to assess expression levels of unigenes by calculating the Transcripts Per Million (TPM) [87]. The differentially expressed unigenes were identified based on log2 (fold change) ≥ 1 or log2 (fold change) ≤ −1 with statistical significance (*p* < 0.05) evaluated by R package edgeR [88].

### 4.5. Proteome Sampling, Sequencing, and Bioinformatics

The microalgal cells of *H. pluvialis* were added with SDT lysate, homogenized, and broken, and boiled in hot water at 100 °C for 10 min. The samples were centrifuged for 15 min at 14,000 g with the supernatant collected. BCA method was used for protein quantification [89]. Filter aided proteome preparation (FASP) was used for enzymatic hydrolysis of proteins [90]. The peptides were desalted by C18 cartridge. After freeze-drying, 0.1% formic acid solution was added for re-dissolution, and the peptides were quantified. The peptide mixture was graded by Agilent 1260 infinity II HPLC system (Agilent Technologies, Santa Clara, CA, USA).

The proteomic sequencing was performed by Hangzhou Lianchuan Biotechnology Co., Ltd. (LC Sciences, Houston, TX, USA). The original mass spectrometry data were combined and analyzed by Spectronaut Pulsar X (version 12, Biognosys AG, Schlieren, Switzerland). TransDecoder (https://github.com/TransDecoder/TransDecoder/, accessed on 16 March 2020) was used to predict the transcript of unigenes to obtain the protein sequences and to establish the spectrum database by Spectronaut Pulsar X (version 12, Biognosys AG, Schlieren, Switzerland). Trypsin enzymolysis was set to allow two missing sites, with the following searching parameters included: (1) fixed modification: carbamidomethyl (c); (2) variable modification: oxidation (m) oxidation; and (3) N-terminal acetylation of acetyl (protein N-term) protein. A total of 1 μg of peptides from each sample was added with iRT peptides to mix and then to inject for separation using Nano-LC. Data were analyzed by online electrospray tandem mass spectrometry. The complete liquid–mass tandem system contained both the liquid system based on the Easy nLC system (Thermo Fisher Scientific, Waltham, MA, USA) and the mass spectrometry system using the Orbitrap Exploris 480 (Thermo Fisher Scientific, Waltham, MA, USA). Buffer solution A solution was 0.1% formic acid aqueous solution and B solution was 0.1% formic acid acetonitrile aqueous solution (80% acetonitrile). The sample was separated by a non-linearly increasing gradient in an analytical column (Nano Technology Column, 18 cm C18 column, with 1.9 μm C18 Resin, Catalog Number 26350-3) at a flow rate of 300 nL/min: 0–1 min, 2% B to 8% B; 1–99 min, 8% B to 27% B; 99–113 min, 27% B to 35% B; 113–117 min, 35% B to 100% B; and 117–120 min, 100% B. The electrospray voltage was set to 2056 V.

Raw data of DIA were processed and analyzed by Spectronaut 14.6 (Biognosys AG, Schlieren, Switzerland) with default settings with the Retention time prediction type set to dynamic iRT. Data extraction was determined by Spectronaut Pulsar X based on the extensive mass calibration. Spectronaut 14.6 was used to determine the ideal extraction window dynamically depending on iRT calibration and gradient stability. Q-value (FDR) cutoff on precursor and protein level was applied at 1%. Decoy generation was set to mutated which was similar to scrambled but only applied a random number of AA position swamps (min = 2, max = length/2). All selected precursors passing the filters were used for quantification. MS2 interference was used to remove most interfering fragment ions except for the 3 least interfering ones. The average top 3 filtered peptides which passed the 1% Q-value cutoff were used to calculate the major group quantities. Differentially expressed proteins were filtered based on Student’s *t*-test (*p* < 0.05 and fold change > 1.5).

GO annotation based on unigenes was performed using Blast2GO [91]. KEGG enrichment analysis was performed based on the KEGG automatic annotation server (KAAS) [92].

### 4.6. Statistical Analysis

All experiments were performed in triplicates to ensure reproducibility. Data were presented as mean ± standard deviation (SD). The paired-samples t-test was performed using ANOVA with the statistically significant difference set at *p* ≤ 0.05.

## 5. Conclusions

In this study, *H. pluvialis* was exposed to high-intensity blue light and SA, resulting in alterations in gene and protein expressions from those derived from exposure to white light of high intensity. Using transcriptomic and proteomic sequencing, we further analyzed the molecular responses in astaxanthin synthesis, fatty acid synthesis, heme synthesis, ROS scavenging pathway, and cell wall synthesis of *H. pluvialis* treated with blue light and SA. On day 2 of the treatment with blue light irradiation, genes showing significant changes in expression were mainly annotated in the pathways of carbon metabolism, while on day 7, the genes of significant regulation were mainly enriched in the pathways related to proteins. The addition of SA led to the enrichment of genes in disease-related pathways. Because low-intensity blue light caused the up-regulation of genes involved in astaxanthin synthesis, while high-intensity blue light led to the down-regulation of genes related to astaxanthin synthesis, indicating that the promotion of astaxanthin synthesis by blue light is probably related to light intensity. Therefore, it is proposed that, in order to improve the production of astaxanthin in *H. pluvialis*, high-intensity white light should be utilized in *H. pluvialis* cultures because the application of high-intensity blue light and SA decreased the expression of genes involved in the astaxanthin biosynthetic pathway, suggesting that the application of high-intensity blue light and SA was not beneficial for maximizing the astaxanthin production. In the cell wall synthesis pathway, the expression of proteins and genes was significantly decreased on day 2 of the treatment with blue light irradiation, whereas the gene expression was significantly increased on day 7. In *H. pluvialis*, the high-intensity blue light led to the significant up-regulation of ROS scavenging genes. Furthermore, blue light also reduced the expression of genes related to fatty acid synthesis. Our results showed that SA significantly down-regulated the ROS scavenging genes and genes involved in heme degradation pathway, suggesting that SA reduced the expression of enzymes related to ROS scavenging, thus promoting the production of astaxanthin by *H. pluvialis*. Moreover, different families of TFs were identified in *H. pluvialis*, induced with different treatment times of blue light irradiation. Our study provides novel insights into the molecular response of *H. pluvialis* to blue light and SA treatments based on both transcriptomic and proteomic analyses.

## Figures and Tables

**Table 1:** Expression of genes encoding three classes of blue light receptors identified in different pairwise comparisons of *Haematococcus pluvialis*.

Class	Unigene ID	B02 vs. W02	B07 vs. W07	B02 vs. N	B07 vs. B02	B07 vs. N	B252 vs. B02	B257 vs. B07
dashCRY	N40695_c3_g2	−0.47	−0.12	−0.54	−0.39	−0.70	−0.17	0.38
dashCRY	N43038_c0_g10	−0.74	−0.49	−1.48	−0.09	−1.34	0.48	0.45
dashCRY	N49636_c1_g1	0.35	−0.33	−4.15	−1.54	−5.46	0.03	1.01
PHOT	N44442_c1_g2	−0.15	−0.03	0.32	−0.48	0.07	0.30	−0.12
PHOT	N50230_c0_g1	−0.32	−0.34	−0.93	−0.68	−1.38	0.14	0.02
CRY	N44179_c1_g4	0.00	−0.41	−1.40	−0.65	−1.83	0.16	−0.03
CRY	N50367_c0_g2	0.21	−0.11	−1.58	−0.97	−2.33	−0.20	0.06

**Table 2:** Top 10 transcription factor (TF) families with number of genes significantly expressed in different pairwise comparisons of *Haematococcus pluvialis*. Symbol “–” indicates the TFs not included in the pairwise comparisons.

Transcription Factor	B02 vs. W02	B07 vs. W07	B257 vs. B07
C2H2	45	30	44
C3H	20	20	16
GNAT	–	13	13
HMG	17	12	16
Jumonji	13	10	–
MYB	21	–	16
MYB-related	18	12	21
SET	13	17	16
SNF2	21	41	38
TRAF	38	57	50
zn-clus	31	43	40

## Data Availability

Raw Illumina sequencing data in this study have been deposited in the Sequence Read Archive (SRA) at the NCBI (http://www.ncbi.nlm.nih.gov/sra accessed on 16 March 2020) under accession number PRJNA766843. The mass spectrometry proteomics data have been deposited to the ProteomeXchange Consortium (http://proteomecentral.proteomexchange.org accessed on 16 March 2020) via the iProX (https://www.iprox.org/ accessed on 16 March 2020) partner repository with the dataset identifier PXD029109 or IPX0003602000.

## Supplementary Materials

The following are available online at https://www.mdpi.com/article/10.3390/md20010001/s1, Table S1: Summary of RNA-seq data in *Haematococcus pluvialis* based on three groups of samples on day 0, day 2, and day 7 after the treatments of white light, blue light, and salicylic acid (SA); Table S2: KEGG enrichment and GO annotation of unigenes identified in *Haematococcus pluvialis*; Table S3: Transcripts and proteins derived from significantly expressed unigenes in *Haematococcus pluvialis*; Table S4: Classification of differentially expressed genes annotated to transcription factors in *Haematococcus pluvialis*, Figure S1: Species distribution of annotated unigenes of *Haematococcus pluvialis*, Figure S2: The length distribution of unigenes of *Haematococcus pluvialis*, Figure S3: Enriched GO terms in three categories (i.e., biological process, cellular component, and molecular function) based on the unigenes with significant changes revealed in the pairwise comparisons of B02 vs. W02 (**A**), B07 vs. W07 (**B**), and B257 vs. B07 (**C**) of *Haematococcus pluvialis*, Figure S4: Enriched KEGG metabolic pathways based on the unigenes with significant changes revealed in the pairwise comparisons of B02 vs. W02 (**A**), B07 vs. W07 (**B**), and B257 vs. B07 (**C**) in *Haematococcus pluvialis*.
