# Supplementary material for: Transcriptomic and Proteomic Characterizations of the Molecular Response to Blue Light and Salicylic Acid in Haematococcus pluvialis
Source: Mar Drugs. 2021 Dec 21;20(1):1. doi: 10.3390/md20010001 (PMC8780009; doi:10.3390/md20010001)
Supplement: Supplementary file 1 [file marinedrugs-20-00001-s001.zip › Figure S4 Enriched KEGG metabolic pathways based on the unigenes with significant changes in Haematococcus pluvialis.pdf]

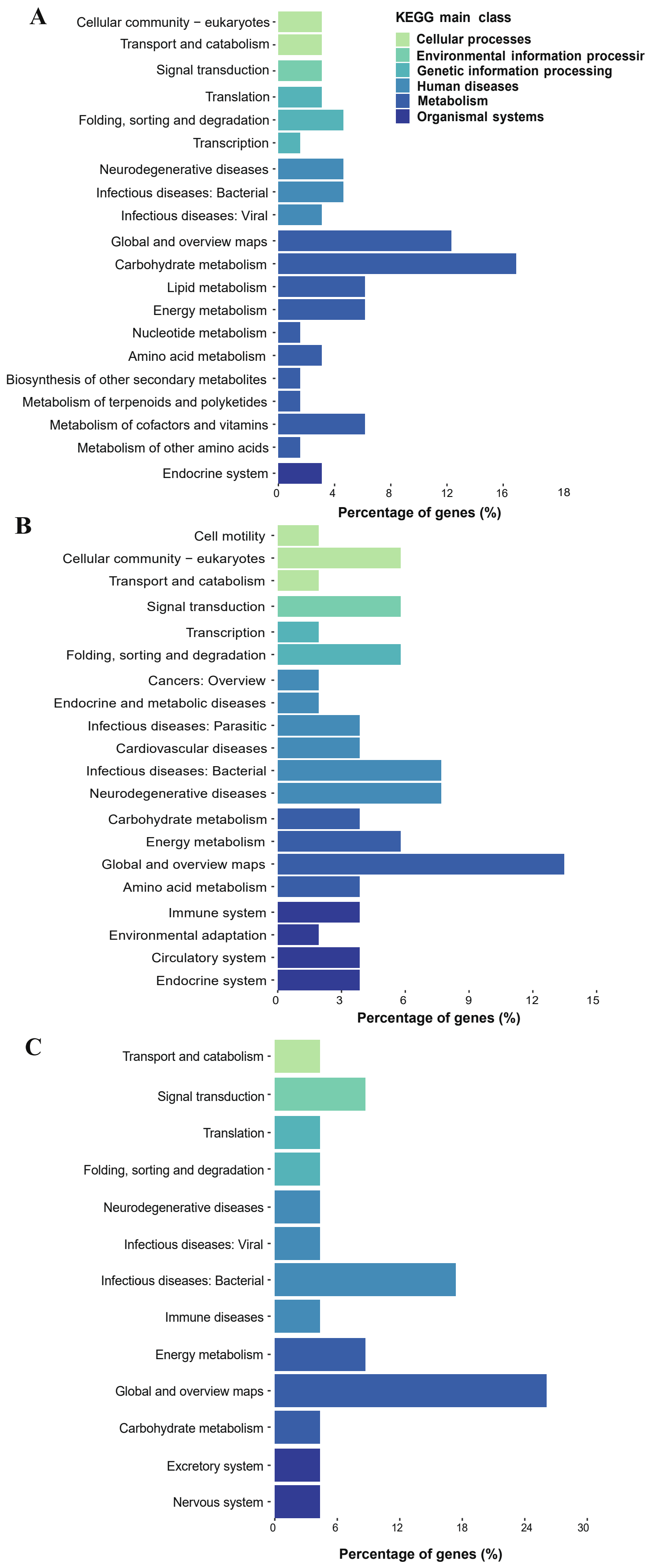

Figure S4. Enriched KEGG metabolic pathways based on the unigenes with significant changes revealed in the pairwise comparisons of B02 vs. W02 (**A**), B07 vs. W07 (**B**), and B257 vs. B07 (**C**) in *Haematococcus pluvialis*.
