# Supplementary material for: Transcriptomic and Proteomic Characterizations of the Molecular Response to Blue Light and Salicylic Acid in Haematococcus pluvialis
Source: Mar Drugs. 2021 Dec 21;20(1):1. doi: 10.3390/md20010001 (PMC8780009; doi:10.3390/md20010001)
Supplement: Supplementary file 1 [file marinedrugs-20-00001-s001.zip › Table S1 Summary of RNA-seq data in Haematococcus pluvialis based on three groups of samples on day 0, day 2, and day 7 after the treatments of white light, blue light, and salicylic acid (SA).pdf]

Table S1. Summary of RNA-seq data in *Haematococcus pluvialis* based on three groups of samples on day 0, day 2, and day 7 after the treatments of white light, blue light, and salicylic acid (SA). "N" indicates sampling on day 0 (white light). B02 and B07 represent samples treated with blue light irradiation for 2 and 7 days without salicylic acid (SA), respectively. W02 and W07 represent samples treated with white light irradiation for 2 and 7 days without SA, respectively. B252 and B257 represent samples treated with blue light irradiation for 2 and 7 days with the addition of SA, respectively. Each treatment was repeated with three replicates indicated as "_1," "_2," and "_3" in the sample names.

Sample	Raw Reads	Clean Reads	Valid%	GC%
Day 0				
N_1	53,024,556	50,073,484	94.43	58.09
N_2	52,384,966	47,745,982	91.14	58.00
N_3	54,712,592	48,657,044	88.93	58.01
Day 2				
W02_1	39,202,360	34,480,996	87.96	57.35
W02_2	50,345,258	46,062,990	91.49	57.29
W02_3	46,365,492	42,373,204	91.39	55.69
B252_1	56,545,752	48,834,448	86.36	57.68
B252_2	47,661,116	41,786,066	87.67	56.75
B252_3	45,954,788	34,585,052	75.26	56.32
B02_1	55,671,946	45,876,098	82.40	56.94
B02_2	57,264,362	49,572,178	86.57	57.50
B02_3	57,085,504	49,425,904	86.58	57.56
Day 7				
W07_1	45,120,414	40,448,046	89.64	57.96
W07_2	36,279,402	32,475,318	89.51	57.63
W07_3	46,378,030	39,765,614	85.74	57.45
B257_1	45,965,390	36,904,362	80.29	55.05
B257_2	45,701,202	40,101,010	87.75	56.57
B257_3	47,725,610	42,774,912	89.63	57.48
B07_1	33,303,774	28,587,214	85.84	56.19
B07_2	41,308,634	34,108,042	82.57	55.60
B07_3	41,476,528	37,653,068	90.78	56.79
