# Supplementary figures and images for: Transcriptomic and Proteomic Characterizations of the Molecular Response to Blue Light and Salicylic Acid in Haematococcus pluvialis
Source: Mar Drugs. 2021 Dec 21;20(1):1. doi: 10.3390/md20010001 (PMC8780009; doi:10.3390/md20010001)

## Species Distribution

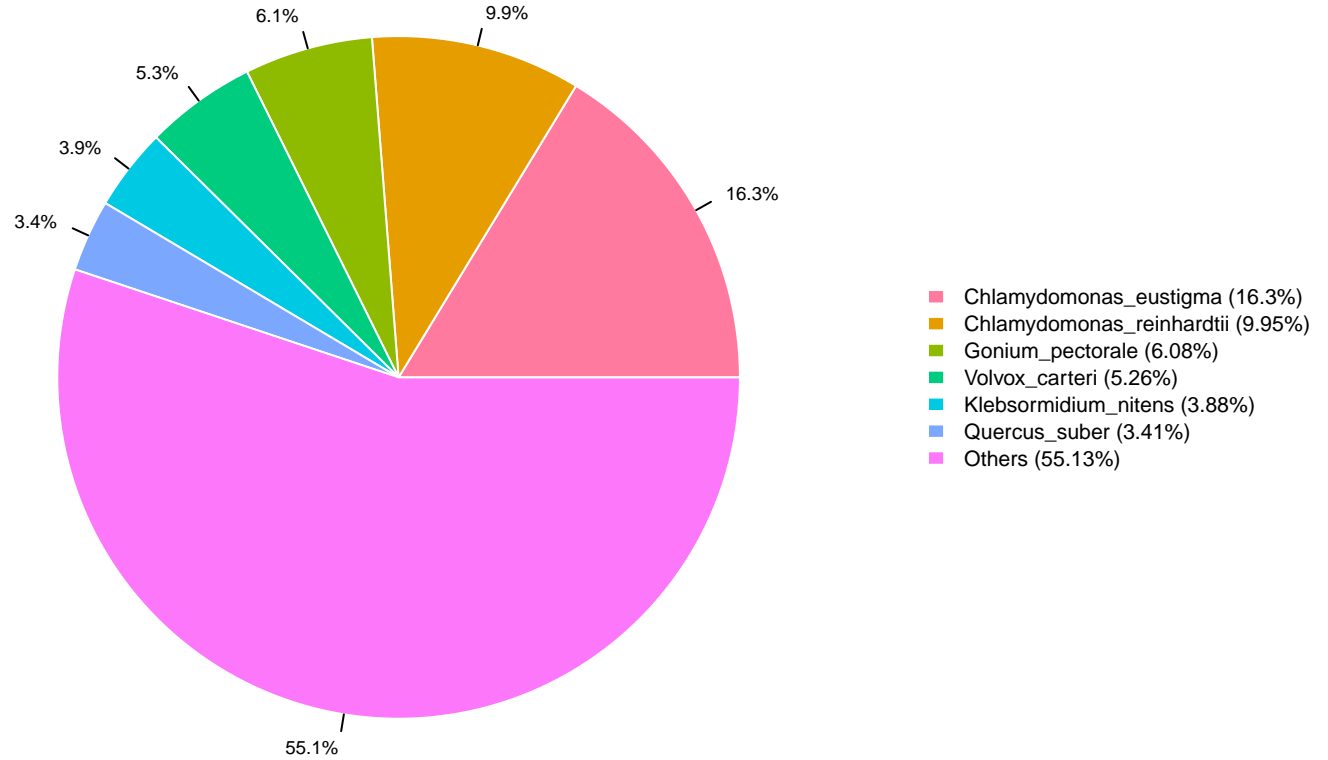

Supplement: Supplementary file 1 [file marinedrugs-20-00001-s001.zip › Figure S1 Species distribution of annotated unigenes of Haematococcus pluvialis.pdf]

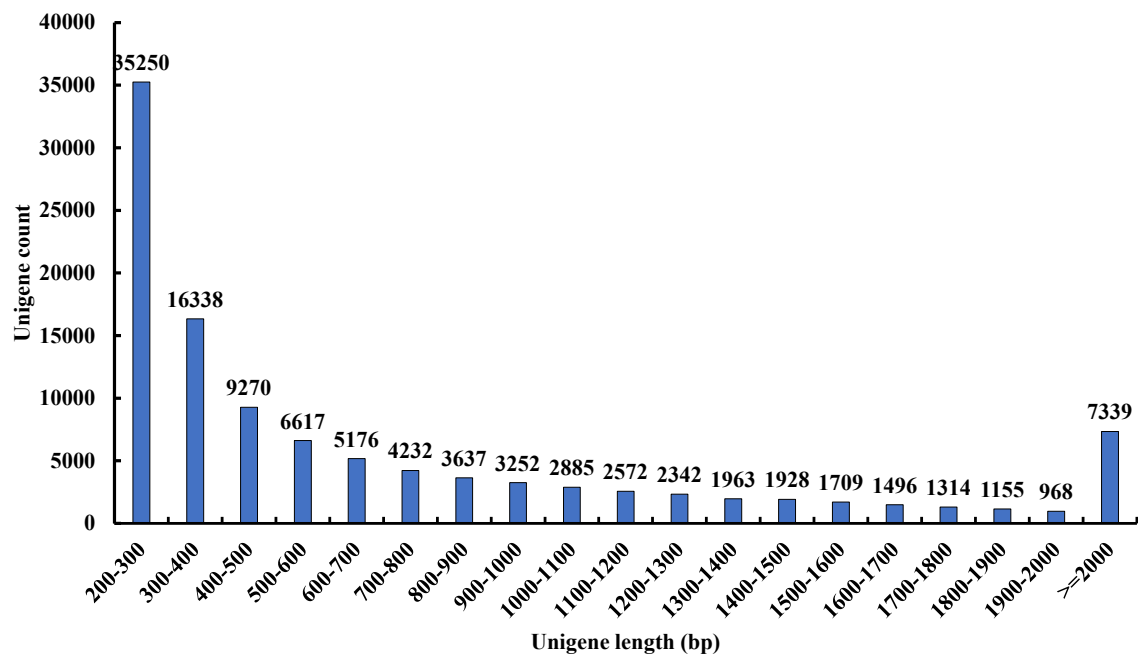

**Figure 1.** The length distribution of unigenes of *Haematococcus pluvialis*.

Supplement: Supplementary file 1 [file marinedrugs-20-00001-s001.zip › Figure S2 The length distribution of unigenes of Haematococcus pluvialis.pdf]
