# Supplementary material for: Transcriptomic and Proteomic Characterizations of the Molecular Response to Blue Light and Salicylic Acid in Haematococcus pluvialis
Source: Mar Drugs. 2021 Dec 21;20(1):1. doi: 10.3390/md20010001 (PMC8780009; doi:10.3390/md20010001)
Supplement: Supplementary file 1 [file Figure S3 Enriched GO terms based on the unigenes with significant changes in Haematococcus pluvialis.pdf › Figure S3 Enriched GO terms based on the unigenes with significant changes in Haematococcus pluvialis.pdf]

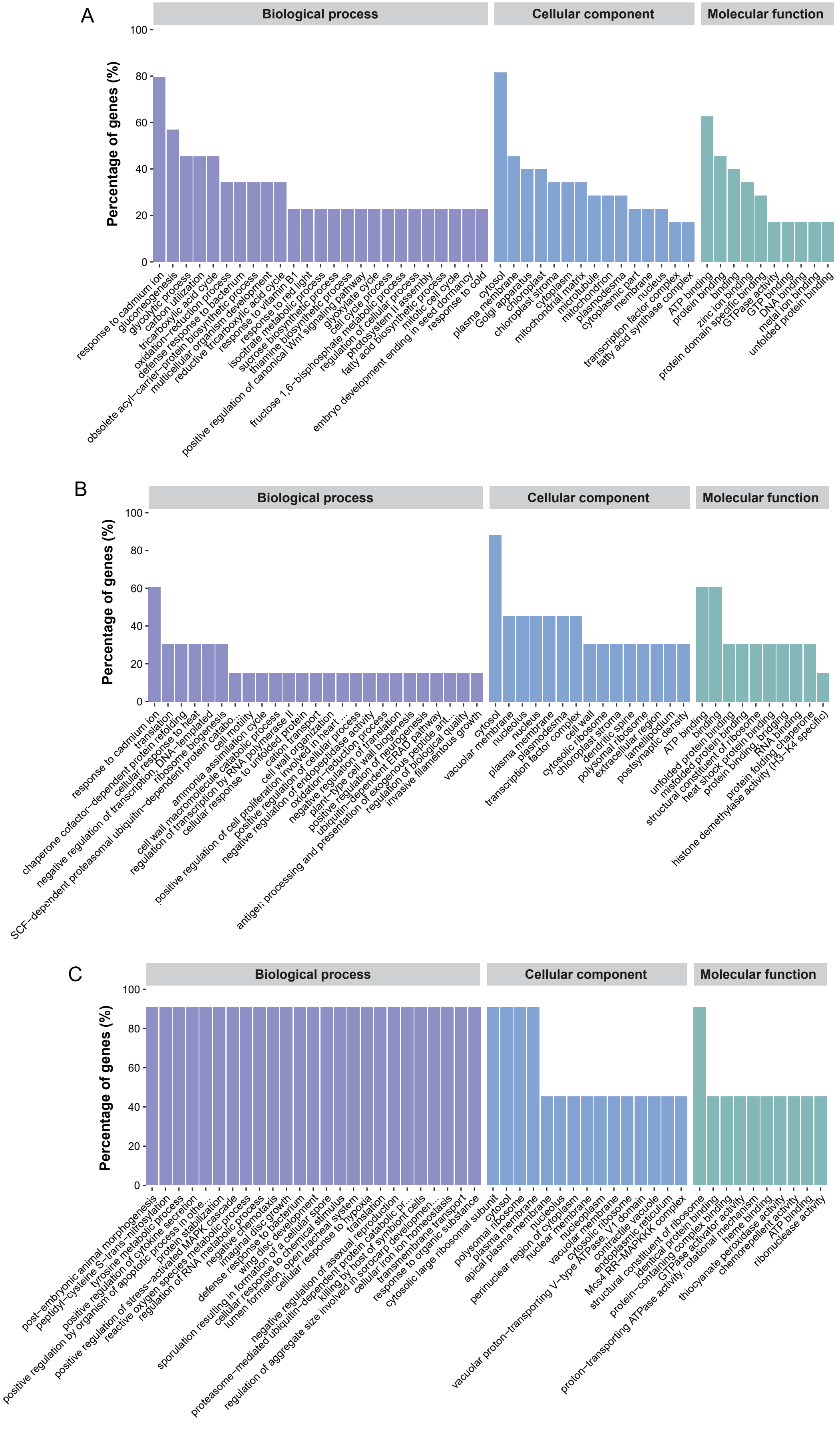

Figure S3. Enriched GO terms in three categories (i.e., biological process, cellular component, and molecular function) based on the unigenes with significant changes revealed in the pairwise comparisons of B02 vs. W02 (**A**), B07 vs. W07 (**B**), and B257 vs. B07 (**C**) of *Haematococcus pluvialis*.
